# Comparative effectiveness of different forms of traditional Chinese medicine for treatment of post-stroke depression: Protocol for network meta-analysis of randomized controlled trials

**DOI:** 10.1097/MD.0000000000016477

**Published:** 2019-07-26

**Authors:** Huiling Chen, Meidan Zhao, Xiuming Li, Yanhong Zhang, Yu Hao, Ergang Xiao, Wenyuan Gao, Hongwu Wang

**Affiliations:** a School of Pharmaceutical Science and Technology, Tianjin University, Nankai; b College of Health Science and Engineering, Tianjin University of Traditional Chinese Medicine, Healthy Industrial Park, Jinghai; c College of Acupuncture and Massage; d Baokang Hospital; e Library, Tianjin University of Traditional Chinese Medicine, Tianjin, China.

**Keywords:** net-meta, protocol, PSD, review

## Abstract

**Background:**

Traditional Chinese medicine (TCM) therapy is effective for post-stroke depression (PSD). TCM therapy encompasses various forms of practices. However, the comparative effectiveness of these therapies is still not clear. Here, we provide a network meta-analysis protocol to compare the effects of different types of TCM therapy on PSD, using both direct and indirect evidence.

**Methods:**

Twelve databases investigation will be conducted through the keywords from their inception to June 1, 2019. At least 2 independent reviewers will identify eligible articles. EndNote X7 software is utilized to manage the literatures and RevMan V.5.3 (The Cochrane Collaboration) software is for data processing throughout the review. The package “netmeta” (version 0.5-0) in R (version 3.0.2, The R Foundation for Statistical Computing) will be used to perform network meta-analysis (NMA). In addition, the overall quality of evidence is evaluated by GRADEPro software, and Cochrane Collaboration Risk of Bias Tool is employed for the methodological quality. Generally speaking, this review protocol is reported according to the preferred reporting items for systematic review and meta-analysis protocols 2015 guidelines.

**Results:**

According to this protocol, it will provide evidence in support of, or against, the hypothesis that TCM therapy for PSD is more effective than pharmacotherapy. The results of this study will also provide evidence on relative efficacy of different forms of TCM. Furthermore, this analysis will show which form(s) of TCM therapy is (are) the most effective.

**Conclusion:**

The results will help PSD doctors and patients choose the treatment regimen which is effective, time-saving and economical.

**PROSPERO registration number:**

CRD42016041594.

## 1 Introduction

Post-stroke depression (PSD) is a debilitating and costly mental disorder, characterized by sustained depressed mood, decreasing interest, and physical fatigue.^[1]^ Approximately 29% patients always suffer from PSD after stroke, and this depression status maintains stable up to 10 years, with a cumulative incidence of 39% to 52% within 5 years of stroke.^[2]^ PSD leads to great functional impairment, poor activities of daily living, cognitive function, and their social function, thus it is a heavy burden for the patients, families, and the society.^[3,4]^ Treatments for PSD mainly include pharmacotherapy, psychotherapy, and traditional Chinese medicine (TCM). People who receive psychological treatments have to ask for professional, bear higher charges and participate the treatment process, which is a challenge for a huge number of people, especially for citizens from low-income regions or busy-working people. Even though available pharmacotherapy has proliferated over the last 20 years, a substantial number of patients either do not respond adequately to these drugs or are unable to tolerate their adverse effects.^[5,6]^ Faced with the shortcomings of the treatment mentioned, TCM therapy may be a potential selection.

TCM, which has been used for thousands of years in China, has been prevalent and considered promising for improving various medical condition, including PSD. TCM encompasses various forms of practices, including acupuncture, moxibustion, Tuina (massage), Chinese herbal medicine (Chinese materia medica), Tai Chi, Qigong, Baduanjin, cupping, Guasha, and dietary therapy.^[7]^ Acupuncture and moxibustion are based on the theory of meridians. They are comprehensive strategy via stimulating specific acupoints or some parts in body surface to promote circulation of Qi and blood.^[8]^ These stimulations have been shown to be beneficial for PSD patients.^[9,10]^ Tuina is one of medical methods which uses various techniques and specific physical activities to prevent and treat diseases in a certain body parts or acupuncture points under the guidance of TCM theory.^[11]^ Chinese herbal medicine is one of the oldest medical treatments in the world. Previous studies have demonstrated the efficacy of Chinese herbal medicine in regulating emotions.^[12–23]^ A review from Wang et al^[24]^ has proven Chinese herbal medicine benefits in improvement of PSD. Research from Tian^[25]^ showed a positive clinical effectiveness through application of Tuina therapy. Combination of Chinese herbal medicine and Tuina therapy has been found to play a significant effect in mood regulation.^[26]^ Tai chi, Qigong, and Baduanjin are some exercises to regulate people's mind and breathing to make the Qi flow more regularly. They showed great potential in promoting depression status without fewer side effect.^[27–29]^ Guasha was widely used by Chinese people. Guasha was reported to be in favor of relieving mood impairment and activities of daily living when combined with acupuncture or cognitive therapy.^[30–32]^ Cupping therapy cured disease through the formed negative pressure and thermal effects in cup.^[33]^ Cupping was helpful to mental status when being added to medicine and psychotherapy treatment.^[34,35]^ The theory of dietary therapy meant the food was divided by different properties and applied to individuals properly.^[36]^ Research about Shi^[37]^ utilized the dietary therapy combined with other therapies, which showed a significant difference compared to control group. Based on the research above mentioned, effects of TCM on PSD have been widely explored.

NMA is a generalization of the traditional pairwise meta-analysis, and it compares all pairs of treatments within a set of treatments for the same disease state (in this case PSD). Along with analyzing direct within-trial comparisons between 2 treatments, the NMA framework enables incorporation of indirect comparisons constructed from 2 trials that have 1 treatment in common.^[38]^

There were some reviews which summarized the effects of TCM on treating PSD.^[39–41]^ However, the existing reviews only contain studies in Chinese. Meanwhile, no network meta-analysis or meta-analysis was found, which analyzed comprehensively the effects of TCM on PSD. Therefore, the aim of this study is to summarize the direct and indirect evidence for TCM and Non-TCM intervention to treatment PSD.

## 2 Methods

### 2.1 Inclusion criteria

1. Language: Published in English and Chinese.
2. Study design: All randomized controlled trials (RCTs). The following items will be excluded: Narrative reviews, letters, meeting abstracts, opinion papers, and any publications without primary data or an explicit description of the methods.
3. Participants: Studies will involve volunteers who are suffering from a previous stroke with a diagnosis of depression according to the criteria of DSM-V, ICD-10, HADS-D, PHQ-9, HDRS, MADRS, SDS, BDI, CES-D.^[42]^ There is no restriction on age or the severity or the stage of the disease. The depression assessment scale is based on grading criteria, and the PSD is divided into light, medium, and severe to describe the severity of depression.
4. Interventions: The treatment group will be given at least 1 of TCM treatment (acupuncture, moxibustion, Tuina [massage], Chinese herbal medicine, Tai Chi, Qigong, Baduanjin, cupping, Guasha, and dietary therapy).
5. Comparators: The control group will be given guideline recommended drug, which includes Fluoxetine hydrochloride, Paroxetine, Venlafaxine, Duloxetine, Sertraline, and citalopram.
6. Outcomes: The primary outcome measure is the number of valid cases. Outcome measures will be assessed using Hamilton rating scale for depression (HAM-D).^[43]^ Valid is defined a ≥50% improvement from baseline on the HAM-D at study end point. The second outcome measure is remission rates. The commonly used definitions of remission is HAM-D ≤7 at study end point.^[44]^
7. Others: The most comprehensive report (eg, the full analysis population) will be used if duplicate publication of the same study or papers published in more than 1 journal.

### 2.2 Exclusion criteria

1. Patients who suffered from serious heart, liver or kidney-related diseases, blood coagulation dysfunction or other complications and other diseases that lead to depression.
2. Patients who are actively participating in other clinical trials or who participated in another clinical trial within the past 90 days.
3. Both crossover and cluster randomized trials will be excluded.

### 2.3 Information sources

Based on the inclusion and exclusion criteria, documents screening, quality assessment of methodology, data extraction were conducted by 2 researchers separately. A preliminary screening (reading of titles and, if need, of abstracts) will be performed in order to include all potentially relevant articles. After the first screening, the final eligibility will be assessed retrieving papers in full text. The following databases will be researched: the Cochrane Central Register of Controlled Trials, PsycINFO, the Cochrane Database of systematic Reviews, CINAHL, Web of science core collection, PubMed, PEDro, EMBASE, the China National Knowledge Infrastructure Database, the Chinese Biomedical Literature Database, the Wanfang database, and the Chinese Scientific Journal Database from their inception to June 1, 2019. We will search (unpublished) completed trials at www.clinicaltrials.gov. We will also search other sources including potential grey literature, conference proceedings, and the reference lists of identified publications and existing systematic reviews. All relevant authors and principal researchers will be contacted to supplement incomplete reports of the original papers or to provide data for unpublished studies.

### 2.4 Search strategy

The main search terms include: acupuncture, moxibustion, Tuina (massage), Chinese herbal medicine, Chinese herbal medicine, Tai Chi, Qigong, Baduanjin (8 section exercises), cupping, Guasha, dietary therapy, body acupuncture, scalp acupuncture, auricular acupuncture, electro acupuncture, acupoints, fire needling, elongated needle and intradermal needling, herb or herbal or Chinese medicine, Chinese Patent Medicine, and so on. A Chinese translation of the same search terms will be used to search in the Chinese databases. The detail of searching strategy for PubMed is shown in Table 1.

**Table 1:**
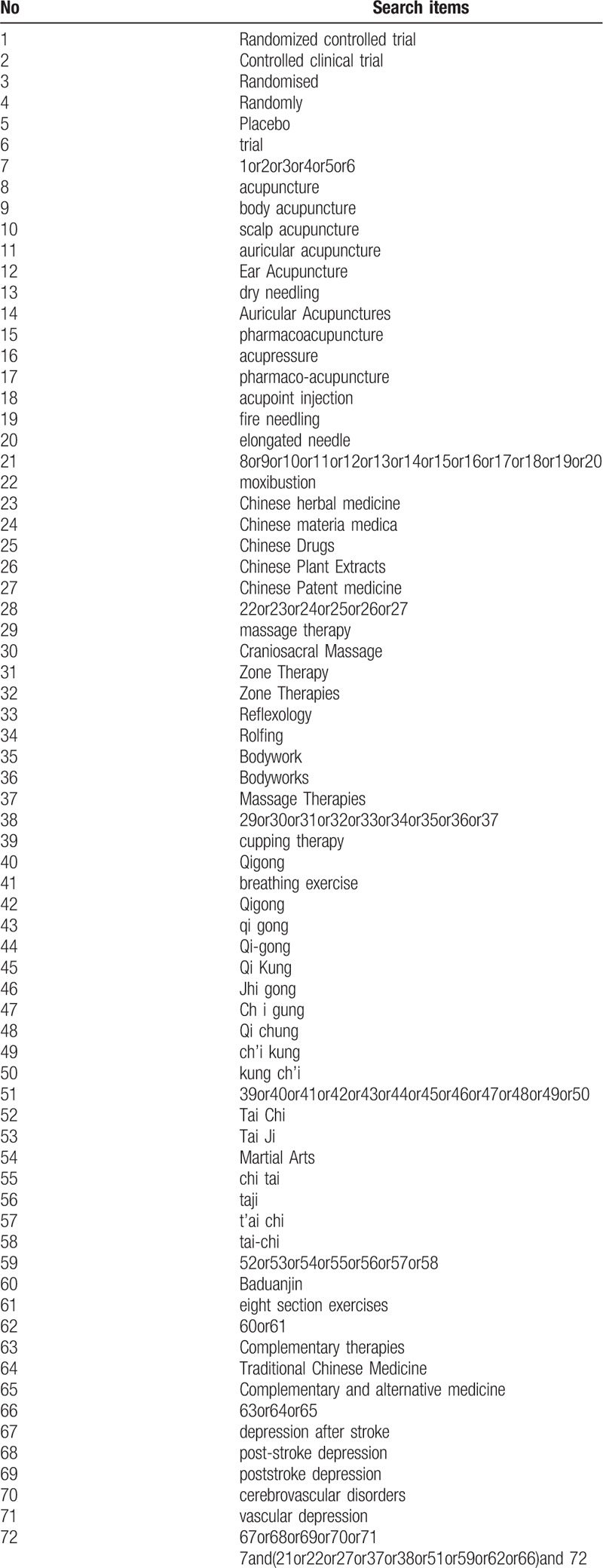
Search strategy used in PUBmed database.

**Figure 1:**
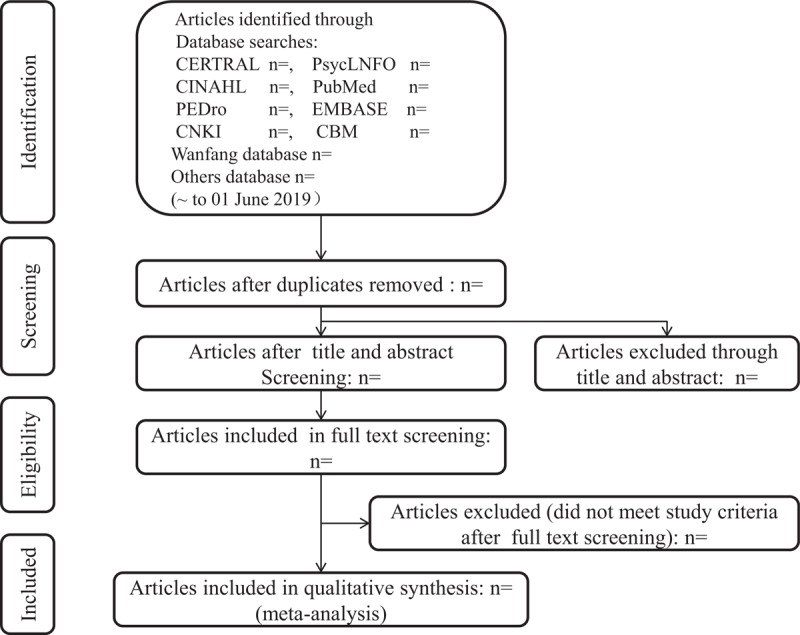
Flow diagram of study.

This review protocol has been prepared according to the preferred reporting items for systematic reviews and meta-analysis protocols (PRISMA-P) 2015^[45]^ statement, and the study results will be presented following the PRISMA flow diagram. The PRISMA-P checklist is submitted in Additional file. Flow chart of the study identification and selection will be shown in Figure 1.

### 2.5 Data extraction

The Data Extraction Template by the Cochrane Consumers and Communication Review Group^[46]^ will be used to extract quantitative data from the literature of quantitative studies. Relevant study characteristics and treatment outcome data will be extracted by 2 investigators (YH and XML). The data extraction will be performed by YH and XML will check the accuracy for the inclusion. Discrepancies will be resolved by discussion or a third investigator (HLC). The data will be extracted as follows:

(1) authors identification;
(2) publication year and the journal;
(3) location and setting; criteria used in diagnosing PSD;
(4) research question/aims;
(5) age range and gender composition;
(6) data collection method;
(7) data analysis method;
(8) study design and random allocation method;
(9) sample size of the intervention and control groups;
(10) duration of intervention and follow-up; inclusive criteria and exclusive criteria;
(11) intervention for the treatment group; intervention for the control group;
(12) course of treatment;
(13) treatment outcome measures obtained;
(14) assessed, the outcome measurement methods/techniques, results, conclusion and funding sources;
(15) treatment-related adverse events.

When there is any uncertainty about the data, we will contact the corresponding author for clarification.

## 3 Statistical

### 3.1 Quality assessment

The risk of bias for each intervention studies will be evaluated using the Cochrane Collaboration Risk of Bias Tool,^[47]^ which includes the following criteria:

(1) random sequence generation or allocation concealment;
(2) blinding of outcome assessment;
(3) selective outcome reporting;
(4) incomplete data assessment.^[48]^

Two reviewers (MDZ and EGX) will be involved in the quality assessment and work independently. Any disagreement will be resolved by discussion or in consultation with HLC.

GRADEPro software will be used to evaluate the overall quality of the evidence. A SOF table will be generated using GRADEPro software (Version3.2 for Windows). This table will evaluate the overall body quality of evidence and incidence of adverse events. In order to achieve this purpose, the method of GRADE criteria will be employed, which includes study limitations, consistency of effect, imprecision, indirectness, and publication bias. The impact of publication bias will be investigated using the funnel plot for asymmetry.

## 4 Data synthesis

### 4.1 Methods for direct treatment comparisons

Initially, it is ensured that the 95% confidence intervals can be derived using a normal distribution. We will perform standard pairwise meta-analyses for every treatment comparison. EndNote X7 software will be used to manage the literature and RevMan V.5.3 (The Cochrane Collaboration) software will be used to process data throughout the review. Heterogeneity will be assessed using Cochran *χ*^2^ test and further quantified using *I*^2^ to decide which effect models are used for meta-analysis.^[47]^

A fixed-effect model will be used to analyze the data if there is no evidence of heterogeneity (*P* ≥ .1, *I*^2^ ≤ 50%). A random-effects model will be used if heterogeneity exists (*P* < .1, *I*^2^ > 50%) and the possible causes from both clinical and methodological will be searched. We will display the results in tables, but do not combine them when heterogeneity is so high (*I*^2^ > 75%).

### 4.2 Subgroup analysis

We will conduct subgroup analysis to determine the evidence for different forms of TCM (acupuncture, Chinese herbal medicine, moxibusition, Tuina therapy, cupping therapy, Qigong, Tai Chi, Baduanjin), different treatment duration (long-term or short term),^[49]^ or different kind of outcome data (post-treatment or difference between pre-post treatment). This also includes subtypes of PSD (different patients conditions), blind method (open trial, single blind for volunteers, double-blind for both volunteers and researchers), quality of evidence (low risk, high risk, unclear of the risk), and age (age <65 and age ≥65) of the volunteers. Additionally, the duration of treatment and combination of treatment (TCM alone or TCM with another treatment) will also be considered. We will conduct subgroup analysis through meta-regression models to quantify the difference between subgroups and test for statistical significance.

### 4.3 Network meta-analysis

We will perform network meta-analysis using the package “netmeta” (version 0.5-0) in R (version 3.0.2, The R Foundation for Statistical Computing).^[50]^ The package uses a novel graph-theory methodology that exploits the analogy between treatment networks and electrical networks to construct a NMA model accounting for the correlated treatment effects in multi-arm trials.^[50]^ The primary analysis, based on a network of treatment method, will be constructed to compare the effect of acupuncture, moxibustion, Tuina (massage), Chinese herbal medicine, mind body therapy (Tai chi, Qigong, and Baduanjin), and others (cupping, Guasha, and dietary therapy). Package “igraph”^[51]^ will be performed to visualize the networks. Bias risk assessment results will be showed by using package “fmsb.”^[52]^ Package “ggplot2”^[53]^ will be performed to visualize the distribution of ranking probability distribution. We will perform sensitivity analyses calculating 95% confidence intervals.

### 4.4 Sensitivity analysis

A sensitivity analysis will be conducted to verify the robustness of the study conclusions and assess the impact of methodological quality, study design, sample size, and the effect of missing data as well as the analysis methods on the result of this review.^[54]^ With sample size of RCTs above 50, sensitivity analysis considering the sample size of the RCTs will be performed. Sensitivity analysis will also be performed on different baselines. Meta-regression analyses will be used on the different follow-up periods.

## 5 Discussion

Owing to moderate cost and acceptable treatment time, TCM is the important treatment for patients with PSD in China. The improvement in PSD symptoms may be gained by using the TCM treatments mentioned above. Since TCM are often used by Chinese PSD patients, synthesizing and comparing the effectiveness on hard clinical outcome parameters becomes more important.

A systematic review of complementary and alternative medicine for depressive disorders^[55]^ was published in 2014. This review included the articles searched out in database from the beginning until May 2013. The above-mentioned article did not include a PSD subgroup meta-analysis and network meta-analysis, whose databases were different from the ones used in this study.

This report describes a planned study to synthesize the views of TCM on the PSD with the aim of providing new interpretations that may facilitate the uptake of recommended treatments, and in turn improve patient care.

Our network meta-analysis will present utility for those patients of PSD, doctors, and policymakers who are regarding use of TCM. Results and conclusions are pending completions of this study. It will also provide evidence on which patient subgroup responds better to TCM form. This conclusion will be derived from a synthesis of quantitative measurement of the overall effect of TCM.

Generally speaking, the study will clarify the existing evidence base regarding the effect on PSD of patient TCM interventions. This conclusion will assist in guiding clinical practice and optimizing the treatment of PSD patients.

If the protocol needs to be amended, there will be a description of the amendment with the reason and the date.

## Acknowledgments

We gratefully acknowledge support from the National Natural Science Foundation of China (grant 81804186) and Basic Research on TCM Health Identification in Tianjin Higher Education Institutions during the Thirteenth Five-Year Plan (Approval No. TD13-5049).

## Author contributions

**Funding acquisition:** Hongwu Wang, Meidan Zhao.

**Methodology:** Huiling Chen, Hongwu Wang.

**Project administration:** Huiling Chen, Wenyuan Gao.

**Resources:** Yu Hao, Yan hong Zhang, Meidan Zhao, Xiuming Li, Ergang Xiao.

**Software:** Ergang Xiao.

**Supervision:** Hongwu Wang.

**Writing – original draft:** Huiling Chen.

**Writing – review and editing:** Huiling Chen.
