# Comparative effectiveness of different forms of traditional Chinese medicine for treatment of post-stroke depression: Protocol for network meta-analysis of randomized controlled trials: Erratum

**DOI:** 10.1097/MD.0000000000017589

**Published:** 2019-10-04

In the article, “Comparative effectiveness of different forms of traditional Chinese medicine for treatment of post-stroke depression: Protocol for network meta-analysis of randomized controlled trials”,^[1]^ which appears in Volume 98, Issue 30 of *Medicine*, Dr. Li Mingxiu's name appeared incorrectly as Xiuming Li.
